# Prevalence of Congenital Heart Disease in Chinese Children With Different Birth Weights and Its Relationship to the Neonatal Birth Weight

**DOI:** 10.3389/fped.2022.828300

**Published:** 2022-05-19

**Authors:** Hui Yan, Bo Zhai, Ruiling Feng, Penggao Wang, Yaodong Zhang, Yiran Wang, Yuwei Hou, Yang Zhou

**Affiliations:** ^1^Henan Provincial Clinical Research Center for Pediatric Diseases, Children’s Hospital Affiliated to Zhengzhou University, Henan Children’s Hospital, Zhengzhou Children’s Hospital, Zhengzhou, China; ^2^Department of Cardiothoracic Surgery, Children’s Hospital Affiliated to Zhengzhou University, Henan Children’s Hospital, Zhengzhou Children’s Hospital, Zhengzhou, China

**Keywords:** congenital heart disease, birth weight, prevalence, special disease database, children

## Abstract

**Objective:**

This study aimed to examine the prevalence and the related risk factors of congenital heart disease (CHD) in children with different birth weights in China and the relationship between the subtypes of CHD and birth weight (BW).

**Methods:**

This study conducted a cross-sectional survey on the data collected in the children’s congenital heart disease database (CHDD) established in China. This database contained data from one Grade A, Level III Children’s Public Hospital in Zhengzhou, Henan. The study included all the children and their parents in the database from 2014 to 2020 as the study subjects, and the missing data were processed by means of imputation. Diagnoses of CHD were coded using the International Classification of Diseases version 10 (ICD-10), and subtypes were classified by the codes Q20 to Q26. We reported the prevalence of CHD based on birth weight and gestational age and analyzed the related risk factors for children with CHD in different birth weight groups and factors for children of the same birth weight groups between the CHD groups and the non-CHD groups. The generalized linear model was used to assess the association between the subtypes of CHD and BW by establishing three adjusting models, and the data were stratified for further analysis by urban-rural and infant gender.

**Results:**

A total of 42,814 children were identified as having CHD among 5,071,799 live children; the overall prevalence of CHD was 8.44 per 1,000 live births during 2014–2020; and the three subtypes with the highest prevalence of CHD were atrial septal defect (ASD) (2.75‰), ventricular septal defect (VSD) (2.57‰), and patent foramen ovale (PFO) (1.12‰). The prevalence of CHD was 18.87‰ in the group with BW <1,500 g, 12.84‰ in the group with BW 1,500–2,500 g, 8.24‰ in the group with BW 2,500–4,000 g, and 4.80‰ in the group with BW ≥4,000 g. The prevalence of CHD was 16.62‰ in the small for gestational age (SGA) group, 6.99‰ in the appropriate for gestational age (AGA) group, and 6.40‰ in the larger for gestational age (LGA) group. Parental factors such as drinking, smoking, viral infections, peri-pregnancy exposure to radioactive substances, low family monthly expenditure, and low Apgar scores at 1 and 5 min were related to the increased risk of CHD in the offspring. Parental supplementation of folic acid and exercise during the peri-pregnancy period could reduce the risk of CHD in the offspring. The results of Model 3 adjusting for confounding variables showed that infants with ASD had a birth weight 461 g lower (95% CI: −1,085, –128), infants with VSD had a birth weight 426 g lower (95% CI: –932, –120), infants with tetralogy of Fallot (TOF) had a birth weight 532 g lower (95% CI: –987, –168), and without classification, infants with CHD had a birth weight 973 g lower (95% CI: –1,502, –204).

**Conclusion:**

In very low birth weight (VLBW) and low birth weight (LBW) infants, CHDs are more prevalent than in the general live-born population. Moreover, some peri-pregnancy factors of parents are closely related to the occurrence of CHD in offspring; different types of heart defects can lead to LBW. Therefore, if the fetus is found to have a heart defect during the prenatal examination, the mother should pay more attention to maintaining weight and ensuring that the fetus is within the normal weight range, thereby increasing the postpartum survival rate, reducing complications, and promoting children’s health.

## Introduction

Congenital heart disease (CHD) is one of the most common congenital malformations. It refers to the abnormal anatomy caused by the formation of the heart and large blood vessels during the development of the embryo, or abnormal development, or channel that should be closed but failed to shut down after birth (1). It was reported that the global average prevalence of CHD at birth was 5–9 per 1,000 births; however, CHD frequencies were as high as 20–40/1,000 in very low birth weight (VLBW) babies weighing less than 1,500 g (2). The etiology of most CHD is not fully understood, but it is likely to involve complex interactions between environmental and genetic factors (3, 4). Similarly, the etiology for low birth weight (LBW) is also exceedingly broad and wide-ranging.

Some studies have shown that there is an association between CHD and LBW, which is a known comorbidity of CHD. A study based on the population of Sudan shows that infants with CHD are 2.6 times more likely to develop LBW than the general Sudanese population (5). CHDs that caused a decrement in birth weight in a descending order of severity are atrial septal defect (721 g/23%), patent ductus arteriosus (669 g/21%), ventricular septal defect (610 g/19%), pulmonary stenosis (548 g/13%), and tetralogy of Fallot (248 g/8%) (5). In addition, the prevalence of CHD in very premature/VLBW infants is higher than that of general live births, and it is independent of other risk factors and causes death and serious complications (6–8).

In China, the prevalence of CHD varies from 7 to 22.9 per 1,000 live births or perinatal infants (9–13), causing serious disease and economic burden to society and families. However, there are few reports on the prevalence of CHD in infants with birth weights less than 2,500 g and more than 4,000 g, and there is a lack of relevant research on the relationship between CHD and LBW. Therefore, it is necessary to conduct a detailed epidemiological investigation of CHD in children with different birth weights in China. This study aimed to determine the prevalence of CHD in very low birth weight infants, low birth weight infants, and giant infants by using the congenital heart disease database (CHDD) established in China containing detailed clinical data. Related risk factors and possible confounding factors were adjusted to further explore the relationship between CHD and birth weight.

## Materials and Methods

### Sources and Subjects

This study conducted a cross-sectional survey on the data collected from the children’s CHDD established in China. The database center was established by a university-affiliated children’s hospital in Henan Province, China (Grade A, Level III Children’s Public Hospital in Zhengzhou, Henan). The CHDD contained childbirth information, such as date of birth, birth weight, feeding method, congenital abnormalities, apgar score of newborn, current health status, and medical history data, and parental sociodemographic information, such as maternal perinatal information (parity, gestational week, and mode of delivery), parents’ socioeconomic factors (age, household registration, occupation, education level, and family monthly expenditure), parents’ lifestyle factors (folic acid supplementation, smoking, drinking, and exercise habits during pregnancy), parents’ exposure to virus, toxic, and harmful substances, and radioactive substances during the perinatal period. The CHDD was connected to the doctor’s medical record system, which could simultaneously obtain the child’s birth information, medical treatment, and record data. The sociodemographic information of the parents was obtained by the investigator through questionnaires and imported into the database in real-time. Data had been collected since 2014; all data were collected prospectively by the hospital data management center, which was equipped with 10 data management specialists for real-time data quality inspections; and then the supervisor completed the review before the data were locked every year.

The study included all the children and their parents in the database from 2014 to 2020 as the study subjects, excluding those with insufficient medical records, transfers after birth, and those who did not want to participate in the study. The study was reviewed and approved by the Ethics Committee of Zhengzhou University, and all respondents signed informed consent. The whole process and all methods of the study were performed in accordance with the relevant guidelines and regulations for the construction of clinical diagnosis and treatment and clinical research ethics review committees.

### Newborn Birth Weight (g) and Diagnosis of Congenital Heart Disease

The birth weight of the newborn was collected by the birth certificate, or the weight was directly measured: after birth, the body was dried, the package was removed, the naked body was placed on the electronic scale, and the stable data were read. The accuracy of the scale was 1 g. When the newborn weighed <1,500 g, it was defined as very low birth weight (VLBW) infant; when the newborn weighed 1,500–2,500 g, it was defined as low birth weight (LBW) infant; when the newborn birth weighed 2,500–4,000 g, it was defined as normal weight infant (NBW); and when the newborn weighed ≥4,000 g, it was defined as macrosomia (14). Referring to the revised report on the birth weight of newborns of different gestational ages in China, the newborns were divided into three categories according to the relationship between birth weight and gestational age: infants were defined as small for gestational age (SGA) when birth weight was below the 10th percentile (P_10_) of the average gestational age; infants with birth weight above the 90th percentile (P_90_) of the same gestational age were defined as larger for gestational age (LGA); and between P_10_ and P_90_ of the same gestational age was appropriate for gestational age (AGA) (15).

In this study, those with positive records of prenatal echocardiography or with abnormal findings in CHD screening were offered neonatal ultrasound screening for CHD. The final diagnosis was based on neonatal echocardiographic findings from a scan and confirmed by the pediatrician. Diagnoses of CHD were coded using the International Classification of Diseases version 10 (ICD-10), and subtypes were classified by the codes Q20–Q26 (16). We defined 13 types of CHD, including atrial septal defect (ASD, Q21.102); ventricular septal defect (VSD, Q21.001); patent foramen ovale (PFO, Q21.103); patent ductus arteriosus (PDA, Q25.001); tetralogy of Fallot (TOF, Q21.300); atrioventricular septal defect (AVSD, Q21.200); pulmonary valve stenosis (PS, Q22.101); tricuspid regurgitation (TR, Q22.801); pulmonary hypertension (PHT, Q25.751); coarctation of the aorta (COA, Q25.300); mitral regurgitation (MR, Q23.300); aortic valve stenosis (AOS, Q23.001); transposition of great vessels (TGA, Q20.302), and other classification was not clear. In the case of multiple diagnoses in one patient, a prespecified hierarchical scheme founded on a consensus-based classification of defect severity was used, by means of which the diagnosis with the worst prognosis was established as the main diagnosis.

### Statistical Analysis

Data were electronically registered. The prevalence of CHD in children with different birth weights was shown as the number of cases per 1,000 births, and any type of CHD was calculated as the total prevalence of CHD per 1,000 births. The continuous variables were statistically described by mean (_
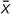
_) and standard deviation (*SD*) and median (*M*) and quartile (*Q*_1_~*Q*_3_); categorical variables were described by composition ratio and rate. For missing values, we used the mean padding method for processing. The *X*^2^ test or, when appropriate, Fisher’s exact test was used to compare the prevalence of various types of congenital heart disease in different birth weight groups and characteristics of children with CHD at different birth weight groups and characteristics of children of the same birth weight groups between CHD and non-CHD groups. Chi-square trend analysis was used to track changes in the prevalence of CHD over time. The means were compared by one-way ANOVA; medians were compared with the Wilcoxon Mann–Whitney rank-sum test. The generalized linear model was used to analyze the relationship between BW and each subtype of CHD, taking BW as the dependent variable, whether each subtype of CHD was diseased as an independent variable, and adjusting possible confounding factors. The research model was as follows: *g(μi) = β_0_ + β_1_x* (whether each subtype of CHD was diseased) + *β_2_x* (adjusted variable) + *εi*. Three models were mainly established as follows: Model 1: no variable was adjusted; Model 2: adjusting fetal sex, gestational age, gravidity, parity, and apgar score at 1 min and 5 min; Model 3: on the basis of Model 2, continuing to adjust the parents’ age, residence (urban and rural), education level, occupation, folic acid supplementation, whether drink, smoke, viral infection, radioactive material exposure, doing physical exercise or not, and family monthly expenditure. And the data were stratified for further analysis by urban-rural and infant gender in the gender-specific model; the baby’s gender was no longer adjusted. These analyses were performed using SAS.9.4 and SPSS25.0; the drawing process was carried out using Microsoft Office 2010 Excel and GraphPad Prism software. *P* values < 0.05 (two-sided) were considered statistically significant, unless indicated otherwise.

## Results

### Patients’ Characteristics and Time Trends of Congenital Heart Disease With Different Birth Weights

From January 2014 to December 2020, a total of 42,814 children were identified as having CHD among 5,071,799 live children. The mean age of children was 6.4 ± 2.2 months. The overall prevalence of CHD was 8.44 per 1,000 live births. The total number of live births with VLBW was 112,560, the number of patients with CHD was 2,124, and the prevalence of CHD was 18.87 per 1,000 live births. The number of LBW newborns was 160,047, the number of patients with CHD was 2,055, and the prevalence of CHD was 12.84 per 1,000 live births. The number of NBW newborns was 4,541,238, the total number of patients with CHD was 37,405, and the prevalence of CHD was 8.24 per 1,000 live births. A total of 256,250 newborns with birth weight ≥4,000 g, the number of children with CHD was 1,230, and the prevalence of CHD was 4.80 per 1,000 live births. The total number of SGA was 786,129, the number of children with CHD was 13,062, and the prevalence of CHD was 16.62 per 1,000 live births. The total number of AGA was 3,950,931, the number of children with CHD was 27,608, and the prevalence of CHD was 6.99 per 1,000 live births. The total number of LGA was 331,875, the number of children with CHD was 2,142, and the prevalence of CHD was 6.40 per 1,000 live births (Table 1).

**TABLE 1:** Prevalence of different subtypes of congenital heart disease by birth weight (average prevalence from 2014 to 2020).

Subgroup	Total		Grouped by birth weight									Grouped by birth weight and gestational age						
			<1500 g		1500∼2500 g		2500∼4000 g		≥4000 g		*P* value	SGA		AGA		LGA		*P* value
	*n*	‰	*n*	‰	*n*	‰	*n*	‰	*n*	‰		*n*	‰	*n*	‰	*n*	‰	
ASD/Q21.102	1994	2.75	57	3.55*^a,c^*	39	1.70*^a,e^*	1635	2.52*^f^*	32	0.87*^c,e,f^*	**<0.001**	289	2.57^➀,➁^	1191	2.11^➀,➂^	52	1.09^➁,➂^	**<0.001**
VSD/Q21.001	1865	2.57	68	4.23*^a,b,c^*	38	1.66*^a^*	1480	2.28*^b,f^*	36	0.98*^c,f^*	**<0.001**	246	2.19^➁^	1085	1.92^➂^	62	1.30^➁,➂^	**0.001**
PFO/Q21.103	813	1.12	17	1.06*^a^*	65	2.84*^a,d,e^*	818	1.26*^d^*	49	1.33*^e^*	**<0.001**	188	1.67^➀^	564	1.00^➀,➂^	88	1.84^➂^	**<0.001**
PDA/Q25.001	587	0.81	13	0.81*^c^*	22	0.96*^e^*	460	0.71*^f^*	5	0.14*^c,e,f^*	**<0.001**	162	1.44^➀,➁^	300	0.53^➀,➂^	9	0.19^➁,➂^	**<0.001**
TOF/Q21.300	379	0.52	92	5.73*^a,b,c^*	47	2.05*^a,d,e^*	278	0.43*^b,d^*	26	0.71*^c,e^*	**<0.001**	377	3.36^➀,➁^	162	0.29^➀,➂^	46	0.96^➁,➂^	**<0.001**
AVSD/Q21.200	294	0.41	35	2.18*^a,b,c^*	17	0.74*^a^*	257	0.40*^b^*	12	0.33*^c^*	**<0.001**	174	1.55^➀,➁^	347	0.61^➀,➂^	10	0.21^➁,➂^	**<0.001**
PS/Q22.101	73	0.10	6	0.37	6	0.26	123	0.19	2	0.05	0.069	80	0.71^➀,➁^	122	0.22^➀^	3	0.06^➂^	**<0.001**
TR/Q22.801	43	0.06	6	0.37	4	0.17	91	0.14	8	0.22	0.074	80	0.71^➀,➁^	79	0.14^➀^	13	0.27^➂^	**<0.001**
PHT/Q25.751	18	0.02	3	0.19*^a^*	47	2.05*^a,d,e^*	69	0.11*^d^*	2	0.05*^e^*	**<0.001**	82	0.73^➀,➁^	39	0.07^➀^	3	0.06^➂^	**<0.001**
COA/Q25.300	15	0.02	2	0.12	3	0.13	37	0.06	0	0.00	0.140	19	0.17^➀^	0	0^➀,➂^	2	0.04^➂^	**<0.001**
MR/Q23.300	12	0.02	2	0.12	2	0.09	32	0.05	0	0.00	0.231	41	0.37^➀,➁^	28	0.05^➀^	1	0.02^➁^	**<0.001**
AOS/Q23.001	9	0.01	1	0.06	1	0.04	27	0.04	4	0.11	0.316	71	0.63^➀,➁^	20	0.04^➀,➂^	15	0.31^➁,➂^	**<0.001**
TGA/Q20.302	7	0.01	1	0.06	1	0.04	21	0.03	0	0.00	0.626	37	0.33^➀,➁^	0	0^➀,➂^	1	0.02^➁,➂^	**<0.001**
Other	7	0.01	0	0.00	2	0.09	16	0.02	1	0.03	0.291	6	0.05^➀^	7	0.01^➀^	1	0.02	**0.017**
Total	6116	8.44	303	18.87*^a,b,c^*	294	12.84*^a,d,e^*	5344	8.24*^b,d,f^*	177	4.80*^c,e,f^*	**<0.001**	1866	16.62^➀,➁^	3944	6.99^➀^	306	6.40^➁^	**<0.001**

*ASD, atrial septal defect; VSD, ventricular septal defect; PFO, patent foramen ovale; PDA, patent ductus arteriosus; TOF, tetralogy of Fallot; AVSD, atrioventricular septal defect; PS, pulmonary valve stenosis; TR, tricuspid regurgitation; PHT, pulmonary hypertension; COA, coarctation of the aorta; MR, mitral regurgitation; AOS, aortic valve stenosis; TGA, transposition of great vessels; Other, classification is not clear. ^a^A difference between the <1,500 g group and the 1,500–2,500 g group; ^b^A difference between the <1,500 g group and the 2,500–4,000 g group; ^c^A difference between the <1,500 g group and the ≥4,000 g group; ^d^A difference between the 1,500–2,500 g group and the 2,500–4,000 g group; ^e^A difference between the 1,500–2,500 g group and the ≥4,000 g group; ^f^A difference between the 2,500–4,000 g group and the ≥4,000 g group; ^➀^A difference between the SGA group and the AGA group; ^➁^A difference between the SGA group and the LGA group; ^➂^A difference between the AGA group and the LGA group. The bold values in the tables represent statistically significant differences.*

**FIGURE 1:**
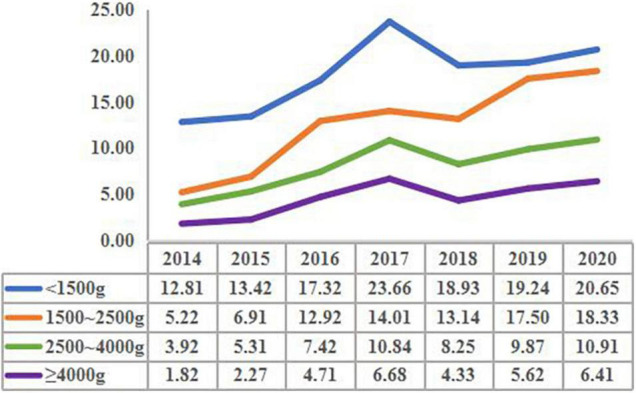
The prevalence of congenital heart disease in children with different birth weights in different years (per 1,000 births).

The prevalence of CHD in the group with birth weight <1,500 g increased from 12.81 per 1,000 births in 2014 to 20.65 per 1,000 births in 2020 (*X*^2^ trend = 125.62, *P* < 0.001). The prevalence of CHD in the group with birth weight 1,500–2,500 g increased from 5.22 per 1,000 births in 2014 to 18.33 per 1,000 births in 2020 (*X*^2^ trend = 98.37, *P* < 0.001). The prevalence of CHD in the group with birth weight 2,500–4,000 g increased from 3.92 per 1,000 births in 2014 to 10.91 per 1,000 births in 2020 (*X*^2^ trend = 85.23, *P* < 0.001). The prevalence of CHD in the group with birth weight ≥4,000 g increased from 1.82 per 1,000 births in 2014 to 6.41 per 1,000 births in 2020 (*X*^2^ trend = 80.19, *P* < 0.001). It was worth noting that the prevalence of CHD in each birth weight group had a peak in 2017 (Figure 1).

### Prevalence of Different Subtypes of Congenital Heart Disease by Birth Weight

Overall, the three subtypes with the highest prevalence of CHD were ASD (2.75‰), VSD (2.57‰), and PFO (1.12‰). In the VLBW group, the three subtypes with the highest prevalence of CHD were TOF (5.73‰), VSD (4.23‰), and ASD (3.55‰). In the LBW group, the three subtypes with the highest prevalence of CHD were PFO (2.84‰), TOF (2.05‰), and PHT (2.05‰). In the NBW group, the three subtypes with the highest prevalence of CHD were ASD (2.52‰), VSD (2.28‰), and PFO (1.26‰). In the macrosomia group, the three subtypes with the highest prevalence of CHD were PFO (1.33‰), VSD (0.98‰), and ASD (0.87‰). Comparing the prevalence of various types of congenital heart disease within different birth weight groups, there were differences in the prevalence of ASD, VSD, PFO, PDA, TOF, AVSD, and PHT among different weight groups. In the group of SGA, the three subtypes with the highest prevalence of CHD were TOF (3.36‰), ASD (2.57‰), and VSD (2.19‰). In the group AGA, the three subtypes with the highest prevalence of CHD were ASD (2.11‰), VSD (1.92‰), and PFO (1.00‰). In the group of LGA, the three subtypes with the highest prevalence of CHD were PFO (1.84‰), VSD (1.30‰), and ASD (1.09‰). Comparing the prevalence of different types of congenital heart disease in different gestational age groups of birth weights, it was found that there were differences in the prevalence among SGA, AGA, and LGA groups (Table 1).

### Risk Factors Related to Congenital Heart Disease

Table 2 shows that the influencing factors of children with CHD in different birth weight groups included maternal characteristics (gestational age, mother’s place of residence, educational levels, folic acid supplementation status, and doing physical exercise during pregnancy and 3 months before pregnancy), paternal characteristics (father’s place of residence, drinking status, smoking status, folic acid supplementation status before pregnancy, and doing physical exercise 3 months before pregnancy), family monthly expenditure, and child factors (apgar score at 1 and 5 min). Mothers tended to be full-term when they gave birth, and the higher the proportion of urban residents and taking folic acid supplements and doing exercise, the higher the education level; the higher the proportion of fathers living in cities and taking folic acid supplements and doing exercise, the lower the proportion of alcohol drinkers and smokers were lower; the higher the monthly family expenditure, the higher the apgar scores of 1 min and 5 min; and the more children with CHD tended to have a normal birth weight.

**TABLE 2:** Characteristics of children with congenital heart disease at different birth weight groups (*N* = 42,814).*

Characteristics	VLBW with CHD (*n* = 2124)	LBW with CHD (*n* = 2055)	NBW with CHD (*n* = 37405)	Macrosomia with CHD (*n* = 1230)	*P* value
Gestational age (week, $\bar{x}\pm s$)	28.5 ± 2.3	33.4 ± 1.5	39.5 ± 1.0	39.8 ± 1.2	**<0.001**
**Maternal place of residence (n(%))**					
Urban	266 (12.5)	399 (19.4)	11334 (30.3)	305 (24.8)	**0.018**
Rural	1858 (87.5)	1656 (80.6)	26071 (69.7)	925 (75.2)	
**Maternal educational levels (n(%))**					
Elementary school and below	601 (28.3)	567 (27.6)	6134 (16.4)	312 (25.4)	**0.035**
Junior high school	1266 (59.6)	1198 (58.3)	6883 (18.4)	426 (34.6)	
High school and secondary school	193 (9.1)	214 (10.4)	18777 (50.2)	387 (31.5)	
University degree and above	64 (3.0)	76 (3.7)	5611 (15.0)	105 (8.5)	
**Maternal folic acid supplementation or not (n(%))^#^**					
Yes	652 (30.7)	783 (38.1)	27867 (74.5)	694 (56.4)	**<0.001**
No	1472 (69.3)	1272 (61.9)	9538 (25.5)	536 (43.6)	
**Mother doing physical exercise during pregnancy (n(%))**					
Yes	280 (13.2)	341 (16.6)	14401 (38.5)	119 (9.7)	**0.037**
No	1844 (86.8)	1714 (83.4)	23004 (61.5)	1111 (90.3)	
**Mother doing physical exercise 3 months before pregnancy (n(%))**					
Yes	335 (15.8)	403 (19.6)	17019 (45.5)	140 (11.4)	**0.030**
No	1789 (84.2)	1652 (80.4)	20386 (54.5)	1090 (88.6)	
**Paternal place of residence (n(%))**					
Urban	316 (14.9)	477 (23.2)	11410 (30.5)	308 (25.0)	**0.018**
Rural	1808 (85.1)	1578 (76.8)	25995 (69.5)	922 (75.0)	
**Father drinks or not (n(%))**					
Yes	1703 (80.2)	1510 (73.5)	15500 (41.4)	657 (53.4)	**0.005**
No	421 (19.8)	545 (26.5)	21905 (58.6)	573 (46.6)	
**Father smokes or not (n(%))**					
Yes	1769 (83.3)	1654 (80.5)	25735 (68.8)	940 (76.4)	**0.038**
No	355 (16.7)	401 (19.5)	11670 (31.2)	290 (23.6)	
**Paternal folic acid supplementation or not before pregnancy (n(%))**					
Yes	406 (19.1)	462 (22.5)	19300 (51.6)	503 (40.9)	**0.016**
No	1718 (80.9)	1593 (77.5)	18105 (48.4)	727 (59.1)	
**Father doing physical exercise 3 months before pregnancy (n(%))**					
Yes	542 (25.5)	748 (36.4)	21620 (57.8)	248 (20.2)	**0.035**
No	1582 (74.5)	1307 (63.6)	15785 (42.2)	982 (79.8)	
Family monthly expenditure (¥, _ 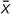 ±s)_	2378.5 ± 362.3	2431.2 ± 276.4	2998.6 ± 430.7	2556.3 ± 379.2	**0.023**
Apgar score at 1 min (M, P_25_–P_75_)	5 (3–6)	5 (4–7)	6 (4–8)	6 (3–8)	**0.002**
Apgar score at 5 min (M, P_25_–P_75_)	7 (5–8)	7 (6–8)	8 (7–9)	8 (6–9)	**0.001**

*Only statistically significant variables are shown; ^#^replenish folic acid for at least 3 months before or during pregnancy. The bold values in the tables represent statistically significant differences.

Further analysis showed that factors that differed between those with and without CHD in the VLBW group included parents’ characteristics (maternal and paternal viral infection, drinking status, radioactive material exposure, folic acid supplementation status, and maternal smokes status), family monthly expenditure, and child factors (apgar score at 1 min). The factors that differed between those with and without CHD in the LBW group included parents’ characteristics (maternal and paternal drinking status, smoking status, radioactive material exposure, folic acid supplementation status, and maternal viral infection), family monthly expenditure, and child factors (apgar score at 1 min and 5 min). The factors that differed between those with and without CHD in the NBW and macrosomia group were identical, including parents’ characteristics (maternal and paternal viral infection, drinking status, smoking status, radioactive material exposure, folic acid supplementation status, and maternal educational levels) and child factors (apgar score at 5 min) (Table 3).

**TABLE 3:** Characteristics of children of the same birth weight between CHD and non-CHD groups.*

A						
**Characteristics**	**VLBW with CHD (*n* = 2124)**	**VLBW with Non-CHD (*n* = 110279)**	***P* value**	**LBW with CHD (*n* = 2055)**	**LBW with Non-CHD (*n* = 158265)**	***P* value**
**Maternal viral infection (n(%))**						
Yes	266 (12.5)	18196 (16.5)	**0.043**	399 (19.4)	37984 (24.0)	**0.015**
No	1858 (87.5)	92083 (83.5)		1656 (80.6)	120281 (76.0)	
**Mother drinking or not (n(%))**						
Yes	200 (9.4)	3419 (3.1)	**<0.001**	154 (7.5)	3640 (2.3)	**<0.001**
No	1924 (90.6)	106860 (96.9)		1901 (92.5)	154625 (97.7)	
**Mother smoking or not (n(%))**						
Yes	164 (7.7)	2757 (2.5)	**<0.001**	115 (5.6)	3165 (2.0)	**0.001**
No	1960 (92.3)	107522 (97.5)		1940 (94.4)	155100 (98.0)	
**Maternal radiation material exposure (n(%))**						
Yes	134 (6.3)	2095 (1.9)	**<0.001**	105 (5.1)	2849 (1.8)	**0.005**
No	1990 (93.7)	108184 (98.1)		1950 (94.9)	155416 (98.2)	
**Maternal folic acid supplementation or not (n(%))^#^**						
Yes	652 (30.7)	61205 (55.5)	**0.016**	783 (38.1)	95750 (60.5)	**0.011**
No	1472 (69.3)	49074 (44.5)		1272 (61.9)	62515 (39.5)	
Paternal viral infection (n(%))						
Yes	316 (14.9)	19519 (17.7)	**0.047**	477 (23.2)	40199 (25.4)	0.058
No	1808 (85.1)	90760 (82.3)		1578 (76.8)	118066 (74.6)	
**Father drinking or not (n(%))**						
Yes	1703 (80.2)	74218 (67.3)	**0.038**	1510 (73.5)	95909 (60.6)	**0.027**
No	421 (19.8)	36061 (32.7)		545 (26.5)	62356 (39.4)	
**Father smoking or not (n(%))**						
Yes	1769 (83.3)	88333 (80.1)	0.079	1654 (80.5)	110786 (70.0)	**0.031**
No	355 (16.7)	21946 (19.9)		401 (19.5)	47479 (30.0)	
**Paternal radiation material exposure (n(%))**						
Yes	89 (4.2)	1434 (1.3)	**0.007**	68 (3.3)	1583 (1.0)	**0.018**
No	2035 (95.8)	108845 (98.7)		1987 (96.7)	156682 (90.0)	
**Paternal folic acid supplementation or not before pregnancy (n(%))**						
Yes	406 (19.1)	31430 (28.5)	**0.031**	462 (22.5)	56501 (35.7)	**0.037**
No	1718 (80.9)	78849 (71.5)		1593 (77.5)	101764 (64.3)	
**Family monthly expenditure (¥,_ 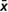 ±s)_**	2378.5 ± 362.3	2597.6 ± 303.1	**0.013**	2431.2 ± 276.4	2771.3 ± 315.9	**0.005**
Apgar score at 1 min (M, P_25_–P_75_)	5 (3–6)	6 (3–8)	**0.027**	5 (4–7)	6 (4–8)	**0.018**
Apgar score at 5 min (M, P_25_–P_75_)	7 (5–8)	7 (4–9)	0.058	7 (6–8)	8 (7–9)	**0.003**

**B**						
**Characteristics**	**NBW with CHD (*n* = 37405)**	**NBW with Non-CHD (*n* = 4503833)**	***P* value**	**Macrosomia with CHD (*n* = 1230)**	**Macrosomia with Non-CHD (*n* = 256608)**	***P* value**

**Maternal viral infection (n(%))**						
Yes	11334 (30.3)	1747487 (38.8)	**0.024**	305 (24.8)	76726 (29.9)	**0.033**
No	26071 (69.7)	2756346 (61.2)		925 (75.2)	179882 (70.1)	
**Maternal educational levels (n(%))**						
Elementary school and below	6134 (16.4)	409849 (9.1)	**<0.001**	312 (25.4)	36182 (14.1)	**<0.001**
Junior high school	6883 (18.4)	463895 (10.3)		426 (34.6)	48499 (18.9)	
High school and secondary school	18777 (50.2)	1819549 (40.4)		387 (31.5)	94432 (36.8)	
University degree and above	5611 (15.0)	1810540 (40.2)		105 (8.5)	77495 (30.2)	
**Mother drinking or not (n(%))**						
Yes	1608 (4.3)	54046 (1.2)	**0.001**	71 (5.8)	5389 (2.1)	**0.005**
No	35797 (95.7)	4449787 (98.8)		1159 (94.2)	251219 (97.9)	
**Mother smoking or not (n(%))**						
Yes	1347 (3.6)	36031 (0.8)	**<0.001**	47 (3.8)	2566 (1.0)	**<0.001**
No	36058 (96.4)	4467802 (99.2)		1183 (96.2)	254042 (90.0)	
**Maternal radiation material exposure (n(%))**						
Yes	1534 (4.1)	40534 (0.9)	**<0.001**	58 (4.7)	3849 (1.5)	**<0.001**
No	35871 (95.9)	4463299 (99.1)		1172 (95.3)	252759 (98.5)	
**Maternal folic acid supplementation or not (n(%))^#^**						
Yes	27867 (74.5)	4062457 (90.2)	**<0.001**	694 (56.4)	183475 (71.5)	**0.001**
No	9538 (25.5)	441376 (9.8)		536 (43.6)	73133 (28.5)	
**Paternal viral infection (n(%))**						
Yes	11410 (30.5)	1801533 (40.0)	**0.021**	308 (25.0)	77752 (30.3)	**0.027**
No	25995 (69.5)	2702300 (60.0)		922 (75.0)	178856 (69.7)	
**Father drinking or not (n(%))**						
Yes	15500 (41.4)	1378173 (30.6)	**0.005**	657 (53.4)	118553 (46.2)	**0.010**
No	21905 (58.6)	3125660 (69.4)		573 (46.6)	138055 (53.8)	
**Father smoking or not (n(%))**						
Yes	25735 (68.8)	2098786 (46.6)	**<0.001**	940 (76.4)	169105 (65.9)	**0.017**
No	11670 (31.2)	2405047 (53.4)		290 (23.6)	87503 (34.1)	
**Paternal radiation material exposure (n(%))**						
Yes	1047 (2.8)	13511 (0.3)	**<0.001**	39 (3.2)	2309 (0.9)	**0.006**
No	36358 (97.2)	4490322 (99.7)		1191 (96.8)	254299 (99.1)	
**Paternal folic acid supplementation or not before pregnancy (n(%))**						
Yes	19300 (51.6)	3121156 (69.3)	**0.013**	503 (40.9)	125225 (48.8)	**0.031**
No	18105 (48.4)	1382677 (30.7)		727 (59.1)	131383 (51.2)	
Apgar score at 5 min (M, P_25_–P_75_)	8 (7–9)	9 (8–10)	**0.001**	8 (7–9)	9 (7–10)	**0.001**

*Only statistically significant variables are shown; ^#^replenish folic acid for at least 3 months before or during pregnancy. The bold values in the tables represent statistically significant differences.

### The Relationship Between Different Subtypes of Congenital Heart Disease and Birth Weight

In the absence of stratified analysis, the ASD, VSD, TOF, and total CHD status had a statistically significant detrimental effect on birth weight in Model 1; after adjusting for some confounding factors (Model 2), similar results were still obtained; and after further adjusting for other confounding factors (Model 3), the results had not changed. On average, infants with ASD had a birth weight 461 g lower (95% CI: –1,085, –128), infants with VSD had a birth weight 426 g lower (95% CI: –932, –120), infants with TOF had a birth weight 532 g lower (95% CI: –987, –168), and without classification, infants with CHD had a birth weight 973 g lower (95% CI: –1,502, –204). After stratifying by gender of children, among boys, multiple models showed that VSD and TOF had significant adverse effects on birth weight. Infants with VSD were associated with a mean loss of birth weight of 467 g (95% CI: –1,052, –117), and infants with TOF were associated with a mean loss of birth weight of 1,056 g (95% CI: –1,424, –537) in Model 3. Among girls, multiple models showed that ASD, TOF, and total CHD had significant adverse effects on birth weight. Infants with ASD were associated with a mean loss of birth weight of 738 g (95% CI: –1,519, –295), infants with TOF were associated with a mean loss of birth weight of 544 g (95% CI: –906, –194), and infants with CHD were associated with a mean loss of birth weight of 812 g (95% CI: –1,359, –285) in Model 3. In addition, infants with PDA were associated with a mean loss of birth weight of 283 g (95% CI: –511, –55) only in Model 3 (Table 4).

**TABLE 4:** Generalized linear model results of the relationship between different subtypes of congenital heart disease and birth weight in children.*

	ASD		VSD		PDA		TOF		Total CHD	
	*β (95%CI)*	*P* value	*β (95%CI)*	*P* value	*β (95%CI)*	*P* value	*β (95%CI)*	*P* value	*β (95%CI)*	*P* value
**The boy**										
**Model 1**	−120 (−201, 392)	0.818	−252 (−827, −30)	**0.005**	−285 (−449, 276)	0.668	−838 (−1321, −397)	**0.038**	237 (−112, 552)	0.394
**Model 2**	−95 (−152, 278)	0.912	−398 (−990, −85)	**0.010**	−175 (−448, 299)	0.795	−934 (−1406, −407)	**0.025**	202 (−243, 577)	0.515
**Model 3**	−86 (−165, 293)	0.907	−467 (−1052, −117)	**0.016**	−124 (−423, 275)	0.541	−1056 (−1424, −537)	**0.007**	170 (−220, 460)	0.278
**The Girl**										
**Model 1**	−502 (−1004, −199)	**0.032**	−180 (−540, 419)	0.584	−152 (−441, 320)	0.738	−372 (−663, −99)	**0.019**	−530 (−983, −125)	**<0.001**
**Model 2**	−680 (−1343, −103)	**0.017**	−149 (−598, 537)	0.721	−168 (−483, 354)	0.761	−527 (−845, −199)	**0.016**	−648 (−1069, −128)	**<0.001**
**Model 3**	−738 (−1519, −295)	**0.009**	−193 (−502, 484)	0.422	−283 (−511, −55)	**0.035**	−544 (−906, −194)	**0.012**	−812 (−1359, −285)	**<0.001**
**Total**										
**Model 1**	−247 (−762, −56)	**0.036**	−270 (−509, −31)	**0.012**	−306 (−891, 391)	0.217	−358 (−778, −93)	**0.034**	−473 (−902, −43)	**<0.001**
**Model 2**	−357 (−965, −107)	**0.033**	−329 (−661, −97)	**0.005**	−274 (−749, 302)	0.233	−459 (−895, −112)	**0.023**	−629 (−1018, −128)	**0.005**
**Model 3**	−461 (−1085, −128)	**0.025**	−426 (−932, −120)	**0.009**	−231 (−628, 393)	0.206	−532 (−987, −168)	**0.018**	−973 (−1502, −204)	**0.001**

*A generalized linear model was used, the dependent variable was birth weight, the independent variable was the type of CHD (ASD, VAD, PDA, and TOF), adjusting for related confounding factors, and β was the model regression coefficient, 95% CI was 95% confidence interval. Model 1: no variable was adjusted; Model 2: adjusting fetal sex, gestational age, gravidity, parity, and apgar score at 1 and 5 min; Model 3: on the basis of Model 2, continuing to adjust the parents’ age, residence (urban and rural), education level, occupation, folic acid supplementation, whether drink, smoke, viral infection, radioactive material exposure, doing physical exercise or not, and family monthly expenditure. In the gender-specific model, the baby’s gender was no longer adjusted. The bold values in the tables represent statistically significant differences.

**FIGURE 2:**
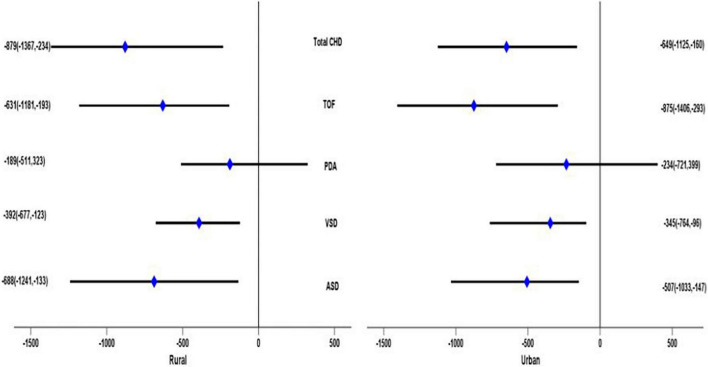
Adjusted β value and 95% CI of the relationship between different subtypes of congenital heart disease and birth weight in children of different places of residence (results of Model 3).

After stratified by residence, children with urban households showed that TOF and total CHD had a significant adverse effect on birth weight under multiple models. On average, infants with TOF had a birth weight 875 g lower (95% CI: –1,406, –293), and infants with CHD had a birth weight 649 g lower (95% CI: –1,125, –160) in Model 3. In addition, infants with ASD had a birth weight 507 g lower (95% CI: 1,033, –147), and infants with VSD had a birth weight 345 g lower (95% CI: –764, –96) only in Model 3. For rural children, the ASD, VSD, TOF, and total CHD status had a statistically significant detrimental effect on birth weight in multiple models. Infants with ASD would cause an average reduction in birth weight of 688 g (95% CI: –1,241, –133), infants with VSD would cause an average weight loss of 392 g (95% CI: –677, –123), infants with TOF would cause an average weight loss of 631 g (95% CI: –1,181, –193), and infants with CHD would cause an average weight loss of 879 g (95% CI: –1,367, –234) in Model 3 (Figure 2).

## Discussion

At present, there have been some reports on the prevalence of CHD in LBW infants and premature infants, and some studies have revealed that there is a certain correlation between CHD and birth weight in the world. However, in China, there are few reports on the prevalence of CHD in VLBW and macrosomia, and there is also a lack of relevant research on the relationship between CHD and birth weight. This study with a large sample size described the epidemiology of CHD in Central Plains of China and could accurately reflect the occurrence of CHD, including the prevalence of some rare subtypes. And we explored the relationship between the subtypes of CHD and birth weight and provided theoretical and practical references for Chinese children to reduce the occurrence of CHD and abnormal birth weight.

We observed that the overall prevalence of CHD was 8.44 per 1,000 live births, and the three subtypes with the highest prevalence of CHD were ASD, VSD, and PFO, which was similar to most of the previous studies (8–10 per 1,000 births at global and local levels) (17–21). The prevalence of CHD in VLBW, LBW, and SGA infants was higher than in other birth weight groups, which was consistent with previous studies (2, 6–8), but the prevalence varied slightly in different regions; the reasons why heterogeneity of CHD prevalence might be differences in study populations, prenatal detection capability, and ascertainment of criteria. The reasons for the increased frequency of CHD in VLBW and SGA infants are still a matter of speculation (22), small septal defects may close spontaneously in utero, and therefore fewer may be apparent in term infants (7). CHD may impair intrauterine growth (23), which justifies the higher frequency of CHD in the VLBW and SGA groups documented by our study. Our research results had observed that, in each birth body reorganization, the prevalence of CHD also increased as the years grew; the increase in the prevalence of CHD in our study might reflect a true increase. The global prevalence of CHD increased from 4.6 per 1,000 live births in 1970–1974 to 9.4 per 1,000 live births in 2010–2017 (17). Increasing trends had also been observed for some specific forms of CHD, such as ASD in the United States and SV, ASD, and TOF in Europe (24, 25). Our results presented that the prevalence of CHD in each birth weight group had a peak in 2017. It might be related to China’s change in the birth policy that opened up the second child in 2016. The parents who gave birth to the second child were generally older, which might lead to an increase in the prevalence of CHD in their offspring.

Our results showed the influencing factors of children with CHD in different birth weight groups, among them, the factors that could be changed included folic acid supplementation before and during pregnancy, more exercise, drinking, and smoking. Folic acid is a water-soluble vitamin, which is indispensable in human metabolism as a coenzyme. Folic acid promotes the development of the fetal nervous system, and the lack of folic acid in the first trimester can lead to neural tube defects in infants (26). Due to the rapid growth and development of the fetus in the second and third trimesters, the demand for folic acid increases sharply; it will be extremely unfavorable to the growth and development of the fetus if the supplement of folic acid is ignored (27). Some studies had suggested that the nutritional status of folic acid during pregnancy was closely related to the growth and development of the fetal (27, 28). One meta-analysis also found that folic acid supplementation during pregnancy was associated with a reduced risk of low birth weight, although only at high doses (29). European and American scholars believed that if pregnant women received adequate nutrition during the development of the fetal nervous system, the heavier the baby’s weight, and the higher the baby’s IQ (28). In addition, good living habits such as physical exercise before and during pregnancy and not drinking or smoking can keep the parents in good health and cultivate healthy fertilized eggs, thereby avoiding the adverse outcome of low birth weight in the offspring (30).

Previous researches had proved that CHD was a multifactorial disease. Our study found that several maternal and paternal factors during pregnancy were associated with an increased risk of their children’s CHD in each birth weight group. These factors included pregnancy viral infection, smoking and drinking, exposure to radioactive substances, folic acid supplementation, exercise, family monthly expenditure, and apgar score. It has been suggested that the key period of heart embryonic development is the second to eighth weeks of pregnancy. Some studies indicated that maternal factors, including viral infection, smoking and drinking, exposure to radioactive substances, or treatment of such infections during the first trimester of pregnancy might precipitate CHD in the children (30, 31). There have been some studies on the relationship between folic acid supplementation during pregnancy and CHD in offspring (32, 33). Our study found that folic acid supplementation could reduce the risk of CHD. Folic acid participates in one-carbon unit metabolism and plays an important role in the process of nucleotide synthesis, amino acid conversion, and methylation (34). Cell proliferation and differentiation during early embryonic development are active, so folic acid metabolism disorders are closely related to early embryonic development abnormalities and birth defects (35). Epidemiological evidence shows that peri-pregnancy supplementation of folic acid or folic acid-containing multivitamins can effectively reduce the risk of CHD, and the key enzyme gene polymorphisms of its metabolic pathways are closely related to CHD (34–37). Folic acid metabolism disorders can lead to the occurrence of hyperhomocysteinemia, which is an independent risk factor for the onset of CHD (38); however, the mechanism of folate metabolism disorders in CHD is still unclear. Some studies have shown that it might cause CHD by affecting the formation and migration of cardiac neural crest cells, hindering DNA synthesis, and interfering with cell proliferation and apoptosis (37–39). Previous studies focused on the influence of maternal perinatal factors on CHD, and our research found that some of the father’s prepregnancy factors also had an impact on the occurrence of CHD. Deng K et al. had showed that fathers who smoked during peri-pregnancy increased the risk of CHD in their offspring, which might be related to the increased probability of sperm chromosome abnormalities and DNA mutations caused by toxic and harmful substances in tobacco, or it might also be related to the increased passive smoking of mothers caused by their fathers’ smoking (40). However, the association between paternal smoking and CHD in offspring needs to be further explored in a large population study. Besides, a father’s alcohol usage and radioactive material intake can increase the risk of CHD in the offspring, which may be largely due to the influence of alcohol and radioactive substances on sperm quality (41, 42).

Our findings clearly demonstrated that infants with CHDs were more likely to be of low birth weight than the general China children. And infants with ASD had a birth weight 461 g lower (95% CI: –1,085, –128), infants with VSD had a birth weight 426 g lower (95% CI: –932, –120), infants with TOF had a birth weight 532 g lower (95% CI: –987, –168), and without classification, infants with CHD had a birth weight 973 g lower (95% CI: –1,502, –204). It closely mirrored previous research done on this topic. Kramer et al. pointed out that the CHD group had significantly lower birth weights, and the decrease in birth weight was distinct only in children with TOF and ASD (43). Other studies showed that the mean birth weight of pure VSD and ASD case subjects was 2.52 and 2.41 kg, which was correspondingly 23% (deficit of 612 g) and 28.6% (deficit of 722 g) less than the control, and the comorbidity of congenital cardiovascular malformations was low birth weight, but the reasons for this association remained obscure (44). It is well established that ventricular and atrial septum are associated with a significant decrease in birth weight (5, 45). Different types of CHD may lead to LBW due to hemodynamics changes, and the specific mechanisms need to be further explored. The types of CHD that causes LBW are not the same in boys and girls, and the specific mechanisms are still unclear.

The advantage of this research is that the survey has been strictly scientifically designed, the data management personnel are of high quality, the data collection work is organized and planned, and the data collected is relatively accurate. This study is with large sample size, and the possible confounding factors are controlled as much as possible during the analysis to increase the reliability of the conclusion. However, there are still some limitations to our study. First of all, we mainly adopt the cross-sectional research method, which cannot verify the causal relationship. Second, we cannot fully monitor all factors that affect birth weight. Peri-pregnancy factors of parents are acquired through recall, which has a certain recall bias. Eventually, some potential confounding factors may not be controlled during the analysis process. This study is an ongoing project, and we will obtain more data on children with continuous improvement of our database system. In this study, we used a database with a large sample size to describe the prevalence of CHD in different birth weights of children in central China from 2014 to 2020, analyzed the parental peri-pregnancy risk factors related to CHD, and explored the relationship between different types of CHD and birth weights. It fills up the gaps in related research in China, and the research results have important reference significance for parents’ peri-pregnancy healthcare, the reduction of CHD, and the improvement of birth weight.

## Conclusion

This study confirms that the prevalence of CHD in VLBW and LBW infants is higher than that of NBW infants in China. Moreover, some peri-pregnancy factors of the parents are closely related to the occurrence of CHD in the offspring; ASD, VSD, and TOF are the main types of CHD that cause LBW in infants. Therefore, prenatal care and other public health efforts such as folic acid supplementation and exercise by parents are expected to reduce the occurrence of CHD and improve the birth weight of the children. And if the fetus is found to have a heart defect during the prenatal examination, the mother should pay more attention to maintaining weight and ensuring that the fetus is within the normal weight range, so as to improve the survival rate after birth, reduce complications, and promote the children’s health.

## Data Availability Statement

The original contributions presented in the study are included in the article/supplementary material, further inquiries can be directed to the corresponding author/s.

## Ethics Statement

The studies involving human participants were reviewed and approved by Ethics Committee of Zhengzhou University. Written informed consent to participate in this study was provided by the participants’ legal guardian/next of kin. Written informed consent was obtained from the individual(s), and minor(s)’ legal guardian/next of kin, for the publication of any potentially identifiable images or data included in this article.

## Author Contributions

HY contributed to conceptualization, methodology, software, statistical analysis of data, writing—original draft preparation, review and editing, investigation, and data curation. BZ contributed to management and coordination responsibility for the research activity planning and execution, data curation, resources, project administration, and validation. RF contributed to formal analysis, data collection and curation, supervision, investigation, and validation. PW contributed to oversight and leadership responsibility for the research activity planning and execution, including mentorship external to the core team, and investigation. YZha contributed to conducting a research and investigation process, specifically performing the data/evidence collection, and investigation and validation. YW contributed to data collection, scrub data, and maintain research data. YH contributed to data collection and data quality control. YZho contributed to visualization, investigation, data curation, supervision, project administration, resources, article revision, and project financial support. All authors contributed to the article and approved the submitted version.

## Conflict of Interest

The authors declare that the research was conducted in the absence of any commercial or financial relationships that could be construed as a potential conflict of interest.

## Publisher’s Note

All claims expressed in this article are solely those of the authors and do not necessarily represent those of their affiliated organizations, or those of the publisher, the editors and the reviewers. Any product that may be evaluated in this article, or claim that may be made by its manufacturer, is not guaranteed or endorsed by the publisher.
